# MicroRNA-629 inhibition suppresses the viability and invasion of non-small cell lung cancer cells by directly targeting RUNX3

**DOI:** 10.3892/mmr.2023.12930

**Published:** 2023-01-03

**Authors:** Bufeng Zhuang, Youshuang Cheng

Mol Med Rep 19: 3933–3940, 2019; DOI: 10.3892/mmr.2019.9990

Following the publication of this paper, it was drawn to the Editors’ attention by a concerned reader that certain of the data shown for the cell invasion assays in Figs. 4D and 5D were strikingly similar to data appearing in different form in other articles by different authors. Owing to the fact that the contentious data in the above article had already been published elsewhere, or were already under consideration for publication, prior to its submission to *Molecular Medicine Reports*, the Editor has decided that this paper should be retracted from the Journal. The authors were asked for an explanation to account for these concerns, but the Editorial Office did not receive a reply. The Editor apologizes to the readership for any inconvenience caused.

